# The role of community capacity building (CCB) in promoting local development: Analyzing leadership in the Hyderabad cantonment community

**DOI:** 10.1371/journal.pone.0352091

**Published:** 2026-06-25

**Authors:** Ghulam Muhammad Abro, Faraz Hussain Kalhoro, Ahmad Alshammari, Bilal Hassan, Ghulam Qadir Abro, Akbar Ali Khan

**Affiliations:** 1 Department of Local Development, Università di Padova, Padova, Italy; 2 Department of Computer Sciences, Faculty of Computing and Information Technology, Northern Border University, Rafha, Kingdom of Saudi Arabia; 3 Faculty of Science and Environment, Department of Computer and Information Sciences, Northumbria University UK London Campus, London, United Kingdom; 4 Department of Statistics, Quaid-i-Azam University, Islamabad, Pakistan; 5 Directorate General Agriculture Extension Sindh Hyderabad, Hyderabad, Sindh, Pakistan; 6 Department of Statistics, Government Postgraduate College, Kohat, Khyber Pakhtunkhwa, Pakistan; Indira Gandhi National Tribal University, INDIA

## Abstract

With leadership positioned as a key tenet, this study investigates the function of Community Capacity Building (CCB) in fostering local development. It combines quantitative survey data from Hyderabad Cantonment, Pakistan, with qualitative interview data using a mixed-methods design. The results show notable deficiencies in women’s leadership participation, resource mobilization, and participatory governance. The study suggests specific tactics to improve leadership for sustainable and inclusive development. The study creates an empirically supported, situation-specific, yet broadly applicable model for grassroots development. Creating localized policy recommendations that address governance deficiencies and integrating leadership roles using both qualitative narratives and quantitative insights are two important contributions. For academics and decision-makers looking to promote participatory governance in urban and semi-urban settings, the results offer useful evidence. It is important to note that findings reflect community perceptions of leadership effectiveness rather than objective performance indicators, and therefore should be interpreted as perception-based evidence rather than causal measurement.

## Introduction

Building community capacity (CCB) has become a key strategy for encouraging sustainable local development, especially in developing nations like Pakistan where governmental institutions frequently struggle with a lack of funding and ineffective implementation systems. Through inclusive leadership, participatory governance, and cooperative decision-making, CCB empowers communities to recognize their developmental challenges and develop localized, sustainable solutions. [1–3]. The local implementation of these frameworks frequently reveals a mismatch between theory and practice, despite the fact that the global discourse on CCB highlights its role in bolstering grassroots governance [4,5]. Authentic involvement is restricted in numerous settings, especially in South Asia where leadership is hierarchical and politically predetermined in most cases [6,7]. Even with a track record of community-based initiatives, Pakistan continues to struggle with the adoption of inclusive leadership concepts that promote transparency, accountability and collaboration [8–10].

Hyderabad Cantonment presents a good case study under this. It illustrates the advantages and disadvantages of implementing CCB principles in a complex governance environment, as it is one of the oldest urban settlements with a diverse socio-political fabric [11]. Using leadership as the primary attribute of CCB, this study bridges the gap between global scholarship and grassroots community development practices [12–14] by relating more general theoretical frameworks to local realities [15–17]. This study explicitly distinguishes between leadership as perceived by community members and leadership as measurable institutional performance. While perception-based assessments provide valuable insight into governance legitimacy and trust, they do not necessarily reflect objective administrative outcomes.

### Problem statement

Hyderabad Cantonment is one of Sindh’s oldest and best-positioned urban areas, but it falls short in terms of gender equity, inclusive governance, and participatory development. Frequently, leadership is short-term, reactive, and disengaged from the needs of the people. Communities suffer from poor service delivery and dwindling trust in institutions as a result of this governance vacuum [18,19].

### Known vs unknown

Prior research has recognized the significance of leadership and community involvement in CCB [6,7,20]. Nevertheless, there is a dearth of empirical research on leadership performance in Pakistani urban settings, especially in cantonment areas where civilian and military administration overlap. Although research around the world places a strong emphasis on accountability and participatory governance, little is known about Hyderabad Cantonment’s particular confluence of politics, history, and community dynamics.

### Aim and hypothesis

The purpose of this study is to investigate how Hyderabad Cantonment’s local development is impacted by leadership, a key CCB attribute. The study’s premise is that poor leadership practices are major obstacles to sustainable development, especially when it comes to the exclusion of women, inadequate resource mobilization, and a lack of accountability. This study closes the gap between CCB theoretical models and empirical data from Pakistani local governance by using a mixed-methods approach.

### Research questions (RQs)

- How far leadership as a characteristic of Community Capacity Building (CCB) contribute to promoting local development in Hyderabad Cantonment?
- How does leadership influence participatory governance and community decision-making?
- What are the leadership contribution in the mobilization of resources and infrastructure development in Hyderabad Cantonment?
- How do local community members perceive the effect of the absence of women leadership on community development?

## Literature review

Theoretical and empirical studies on CCB, leadership, and participatory governance are summarized in this section. Six strands make up its thematic organization: (1) CCB definitions and frameworks; (2) leadership as a foundational element of development; (3) participatory governance; (4) gender and leadership; (5) resource mobilization and collaboration; and (6) global and local perspectives.

### Defining community capacity building

A common definition of CCB is the process through which communities gather institutional mechanisms, resources, and skills to solve problems collectively. [4] conceived CCB as a local development strategy as well as a collection of traits (like trust, leadership, and participation). According to [5], aspects like organizational capacity, resource accessibility, and citizen participation are essential.

### Leadership as a cornerstone of development

CCB has continuously been identified as being driven by leadership. [16] asserts that attaining sustainable governance results requires leadership development. Leadership that is founded upon inclusiveness and transparency enhances trust, cooperation, and civic engagement [8,9,17]. Effective leadership has been demonstrated to promote community resilience and hasten the adoption of new policies in developing contexts [7]. The recent study in the field of public administration strengthens even more the precision that modern local governments rely more and more on adaptive leadership that would be in a position to deal with multi-level institutional coordination and community demands especially in the context of decentralized governance [21].

### Participatory governance

The focus of participatory governance is on decision-making that involves citizens at every level of preparation, execution, and oversight. According to academics [20], participatory approaches boost local institutions’ legitimacy in addition to their efficacy. Weak participatory structures frequently lead to elite capture and marginalized groups’ exclusion in the South Asian context. Recent global evidence demonstrates a renewed expansion of participatory governance mechanisms, particularly participatory budgeting initiatives. For example, in 2024 multiple cities worldwide expanded citizen-led budgeting processes, and the European Union formally recognized participatory budgeting as a pathway to inclusive and transparent governance, indicating growing institutional validation of participatory decision-making systems [22]. Additionally, contemporary research on community-based participatory research (CBPR) emphasizes collaborative governance frameworks where community stakeholders actively co-produce knowledge, shape research agendas, and participate in implementation. Such approaches strengthen legitimacy, improve policy responsiveness, and enhance community trust in governance institutions [23].

### Gender and leadership

Gendered leadership perspectives remain a very vital aspect of CCB. Institutional, cultural and religious restrictions all tend to restrict the leadership opportunities of women. Nonetheless, as revealed by the authors of this paper, women inclusion in the world of leadership has far established better community development outcomes. A paradox that can be noted in the literature is that despite the willingness to assist female leaders in communities, some underlying systemic barriers persist [10,24].

### Resource mobilization and collaboration

In the case of CCB, there is the need to mobilize financial, human, and institutional resources. Communities, according to research conducted by [3] and [25] still survive on paltry government funding without any strategic alliances. Government initiatives can be supported by businesses, NGOs, and religious leaders, yet collaboration often fails, due to the lack of trust or ineffective planning.

### Global and local perspectives

To achieve sustainable development objectives, the importance of capacity building in a global sense is highlighted by such institutions as UN and OECD [11,26,27]. Nevertheless, the accountability system, political favors, and institutional silos are obstacles to these efforts in Pakistan. Cantonment areas are special because of their dual administration and have not been seriously studied in empirical research works. It is this gap that makes the present study justified. Recent research also underlines the fact that perception-driven governance appraisals tend to go off track with administrative performance indicators, especially in developing urban settings, in which informal institutions dictate mass opinion. This brings out a critical review of the role of conjoining subjective and objective measures in determining leadership effectiveness, which supports the methodological applicability of mixed-method methodologies.

## Methodology

The methodology to be used in this study is aimed at having a systematic study of the role of leadership as the main element of Community Capacity Building (CCB) in the Hyderabad cantonment. It was believed that a mixed-methods approach was the most appropriate to reflect most of the quantitative trends of the community perceptions and the finer, qualitative dimensions of leadership, due to the complexity of community governance. This section describes the research design, data sources, sampling plan, outcomes of interest and the methods of analysis.

### Study area

The study happened in Hyderabad Cantonment, which is situated in Hyderabad, the eighth biggest city of Pakistan. Hyderabad is one of the ancient urban settlements in the country and its history dates back to seventeenth century. It is a rich historical and urban heritage which makes it an important case study in examining the issue of community development and governance matters.

The area of the cantonment under the specific study has a population of about 99,767 people and covers an area of 12.14 square kilometers. The region is socially heterogeneous, showing the obvious ethnic stratification having such significant communities as Muhajirs, Sindhis, or Pathans. This ethnic heterogeneity offers some opportunities as well as challenges to participatory governance and leadership as Hyderabad Cantonment is an interesting site to study how the Community Capacity Building (CCB) contributes to the inclusive development of a local area.

### Research design

A convergent mixed-methods design is used in this study, which combines quantitative surveys and qualitative interviews. While the quantitative strand examines generalizability, the qualitative strand documents lived experiences.

### Data source

Information was gathered from Hyderabad Cantonment, a Sindhi neighborhood with a diverse population. It is a special case for researching leadership and governance because of its administrative structure and colonial past.

### Population and sampling technique

For the qualitative part of the study, 13 participants were selected for detailed interviews using purposive sampling, focusing on residents of Hyderabad Cantonment shown in from diverse backgrounds, including local residents, businessmen, current and ex- community leaders and retired employee from administration. This diversity ensured a broad range of perspectives on local leadership.

For the quantitative part, a sample of 50 respondents was also selected through snowball sampling focusing on the background of the respondents who are literate, aware about the key concepts of local development and community participation. Such mix of samples makes it possible to have a depth and a broader perception of the research problem.

The sample is rather small and not probabilistic, but it suits the purposes of exploratory mixed-method research due to the creation of contextual understanding and not statistical generalization. Nonetheless, the sampling strategy and gender division can be a weakness in representativeness, and results must be taken with caution.

### Ethical considerations

Ethical approval to conduct this study was formally obtained from the Military Estate Office, Hyderabad, vide letter No. H-10/4/PF/I dated 05/Nov/2024. Prior to data collection, respondents were informed about the aims and objectives of the research, and their voluntary participation was ensured through informed consent procedures. Participants were assured that all information provided would remain confidential and would be used strictly for academic purposes only. The identity and personal information of respondents were kept anonymous throughout the study. Additional ethical and inclusivity-related information has been provided in the [CTRL U+0091]Inclusivity in Global Research Questionnaire[CTRL U+0092] submitted as a supplementary file.

### Outcomes

The key outcomes of interest are (1) Perceived effectiveness of leadership, (2) inclusivity of governance, (3) gender representation, (4) stakeholder collaboration and (5) trust in leadership.

### Statistical analysis

The descriptive statistics were used to summarize the perceptions, and graphical tools were used to visualize. Inferential statistical tests were used only for exploratory interpretation due to the relatively small and non-probability sample. Therefore, statistically significant findings should not be interpreted as evidence of causality or population-level generalization, but rather as indicative patterns requiring further validation through larger probabilistic studies.

## Results

The results are made in two chains: qualitative narration and quantitative survey data.

### Leadership accessibility, representation, and service orientation

The thematic analysis shows that leadership in Hyderabad Cantonment is generally seen as available, representative and service based. The concept of accessibility turned out to be the cornerstone of effective leadership, with the participants stating that visible and accessible leaders would promote a healthier relationship within the community and responsiveness to local issues. Nonetheless, accessibility is not a standalone factor; the respondents also pointed out that to achieve long-term sustainability and prevent burnout, sustainable leadership involvement should be guided by structured communication channels, boundary setting, and efficient time management.

Representation and advocacy form the other key aspect of leadership performance. Interviewees referred to leaders as freemen who are in charge of communicating community issues to the government. However, the position is quite complicated per se due to the heterogeneity and even contradiction of the needs of the community. Leaders are supposed to juggle between competing priorities, e.g., infrastructure development, social welfare, and environmental protection and they need the ability to negotiate and be tactical in their choices. Lack of control over these conflicting demands could undermine perceived legitimacy. Leadership was also perceived as a functional one and its participants focused on problem-solving and service delivery. Good leaders were supposed to solve local problems, properly distribute limited resources, and act on the urgent needs of the population.

### Perceptions of leadership roles in community development

Although the role of leadership in enhancing infrastructure has been acknowledged, there has been a mixed perception of the effectiveness of leadership by the community and in worst cases, a reproachful view. The respondents admitted that there are noticeable developments in the roads, drain and water facilities which means that the leaders have shown the ability to implement development projects. These successes support the belief that leadership is critical in improving the physical living conditions. Nevertheless, respondents also mentioned that the priorities of leadership are usually disproportionately oriented towards infrastructural outputs, usually at the cost of wider societal concerns. The barriers in administration and a lack of co-ordination with the higher authorities were often mentioned as the barriers to the ability of leaders to respond to the urgent community needs. Such structural limitations can limit deliverance of full developmental results by leaders, thus creating frustrations among the residents.

Participants emphasized that development programs supported by residents were more sustainable because they fostered ownership and shared responsibility. However, equitable participation remained limited, particularly among marginalized groups.

### Participatory governance and dynamics of decision-making

The results show incongruencies in participatory governance practices. The members of the community shared different opinions about the responsiveness of leaders: some of them saw the latter as responsive and communicative, others as irresponsible or unresponsive. This discrepancy implies that the performance of leadership is not even and that it might be contingent on the leadership style, communications practice and social expectations.

Another reoccurring issue was the drop in engagement of the leader after elections. Most of the respondents mentioned that during the election seasons the leaders are very accessible to the public and then they become unavailable at a later time. Post-election disengagement was perceived to weaken trust, accountability, and continuity in governance. This means that in order to have continuity in governance processes it is imperative that there is sustained interaction between the leaders and the constituents to sustain their legitimacy.

It was also deemed to be inconsistent with inclusion in decision-making. Some residents felt that their views were regarded, but other people thought that decisions were made in a closed door manner. The participants highlighted that participation in decision-making will increase empowerment and effectiveness in policy. Nevertheless, the power structure, self-interest, and institutionalized secrecy usually tend to be obstacles to actual engagement, unveiling institutional obstacles to participatory governance.

### Inclusive governance and representation

Another important but undeveloped aspect of leadership practice became inclusive governance. Respondents reported uneven inclusion in decision-making due to administrative constraints and limited access to information. Inequalities in the representation of communal interests were also brought up. Some respondents were of the view that occasionally, leaders adopt the interests of political allies or individual supporters when making decisions and this issue may undermine social cohesion and trust towards governance institutions. Elective representation creates a risk of marginalization of some groups and compromises the validity of development programs.

It was found that stakeholder collaboration was a potentially transformative method of inclusive governance. Respondents identified the roles that the government officials, religious leaders, educators, and civil society actors in enhancing the strength of decision-making processes. Nevertheless, conflict amongst these actors can occur because the actors have conflicting interests, which implies that there should be proper coordination and conflict-management practices in order to achieve the positive outcomes of multi-stakeholder engagement.

### Accountability, trust and leadership responsiveness

Responsiveness and accountability were cited as the crucial qualities of the legitimate leadership on multiple occasions. The participants also stressed that trust to the leaders is directly connected with their capacity to solve societal issues and show integrity. Once the leadership performance falls short of expectations by its population, trust will decay at a fast rate and will limit the cooperation levels among citizens and undermine the governance performance.

The formal accountability mechanisms were also seen as not very effective especially since the local leaders do not have direct control over the financial resources. Although the institutional oversight structures are in place, the respondents mentioned that the effectiveness of these structures is dependent on the quality of implementation and enforcement. Lack of good accountability systems can thus lessen the motivation of the leaders to ensure high levels of performance.

Even though the responsibility to the people is not direct, elections were considered as an indirect means upon which a community can judge the work of the leadership. It has been observed that the availability of communication channels and transparency increases public confidence, which indicates that responsiveness is a major factor in the perceived accountability.

The capacity of civic groups to mobilize their resources and inspire fellow citizens to back their political strategies.

### Resource mobilization capacity

The ability of civic groups to mobilize their resources and encourage other citizens to support their political strategies. The mobilization of resources was one of the key points of concern. There was an indication by many participants that there is enough resource that is not being put to good use because there is a lack of leadership initiative. Limited attempts to attract external financing, make partnerships, or seek creative methods of financing were often mentioned by the respondents. This was seen as inability to take initiative and this was viewed as a limitation to the development.

Leaders are known to have several problems in accessing resources that may include bureaucracy, political restrictions and lack of technical know-how. Such challenges limit their power to execute development plans. According to interviewees, effective leaders should take the initiative to bridge networks with both governmental and private organizations in order to increase the availability of resources.

Another relevant additional resource was found to be community contributions. Development efforts can be enhanced with financial, material and voluntary assistance of residents to enhance sustainability. Participants, however, noted that these contributions are usually not optimally used because of lack of coordination meaning that there exists a disparity between the potential and actual resource mobilization capacity.

### Women leadership and gender Representation

The discussion shows warning acceptance of female leadership involvement as well as the prevalence of structural and cultural obstacles. Despite the lack of opposition to women in leadership positions, most respondents were not sure about their effectiveness which was mainly caused by lacking role models. This points out that the attitude towards leadership by women is still guided by the historical trends and not evidence at hand.

The respondents admitted that women are restricted by social norms and traditional expectations to be able to pursue leadership in society. This hinders the access to decision making rooms by women as well as their participation in the governance processes. Such structural limitations are the factors that cause gender gaps in leadership.

In spite of these issues, most of the interviewees saw potential of women in leadership and pointed out that their involvement can improve development outcomes. Women were explained as an honored and respected community member whose participation can help make the project implementation process easier and the conflict less. Respondents emphasized that the environment should be supportive in the institutions and represented in a greater number to allow the women to give contributions. These results indicate that there is conceptual acceptance on the issue of women leadership, but structural reforms would be necessary so as to put the conceptual acceptance into practical participation.

These findings provide a detailed insight into the relationship and structural barriers that prevail in the ecosystem of leadership in Hyderabad Cantonment. They highlight the urgency of the reform, inclusivity and active participation to bring back confidence and build successful community capacity building.

### Survey-based evidence: Quantitative results

The quantitative part of the study incorporated 50 survey respondents who present a diverse spectrum of opinions within Hyderabad Cantonment. Snowball sampling was used to select the respondents. This was aimed at quantitatively assessing the perceptions of the community regarding gender representation, stakeholder cooperation, participatory governance, and leadership effectiveness. The qualitative themes were used to interpret the results that had been analyzed with SPSS and R.

#### Demographics and representation.

The Table 1 shows population composition indicates that the majority of the population comprises the working-age male adults who are better than secondary and up to the undergraduate degree holders. This sample profile provides an insight into the opinion of individuals who are usually part of civic, economic, and community activities.

**Table 1:** Demographic profile of survey respondents.

Gender	90% Male, 10% Female
**Age Distribution**	82% aged 26–60 years
**Education**	76% with secondary/undergraduate degrees
**Employment**	56% self-employed

All figures referenced below are interpreted only where analytically necessary to support key findings, in accordance with reporting standards that prioritize interpretive relevance over graphical quantity.

### Graphical representation of key research findings

#### Perceived effectiveness of current local leadership in delivering development outcomes.

**Fig 1:**
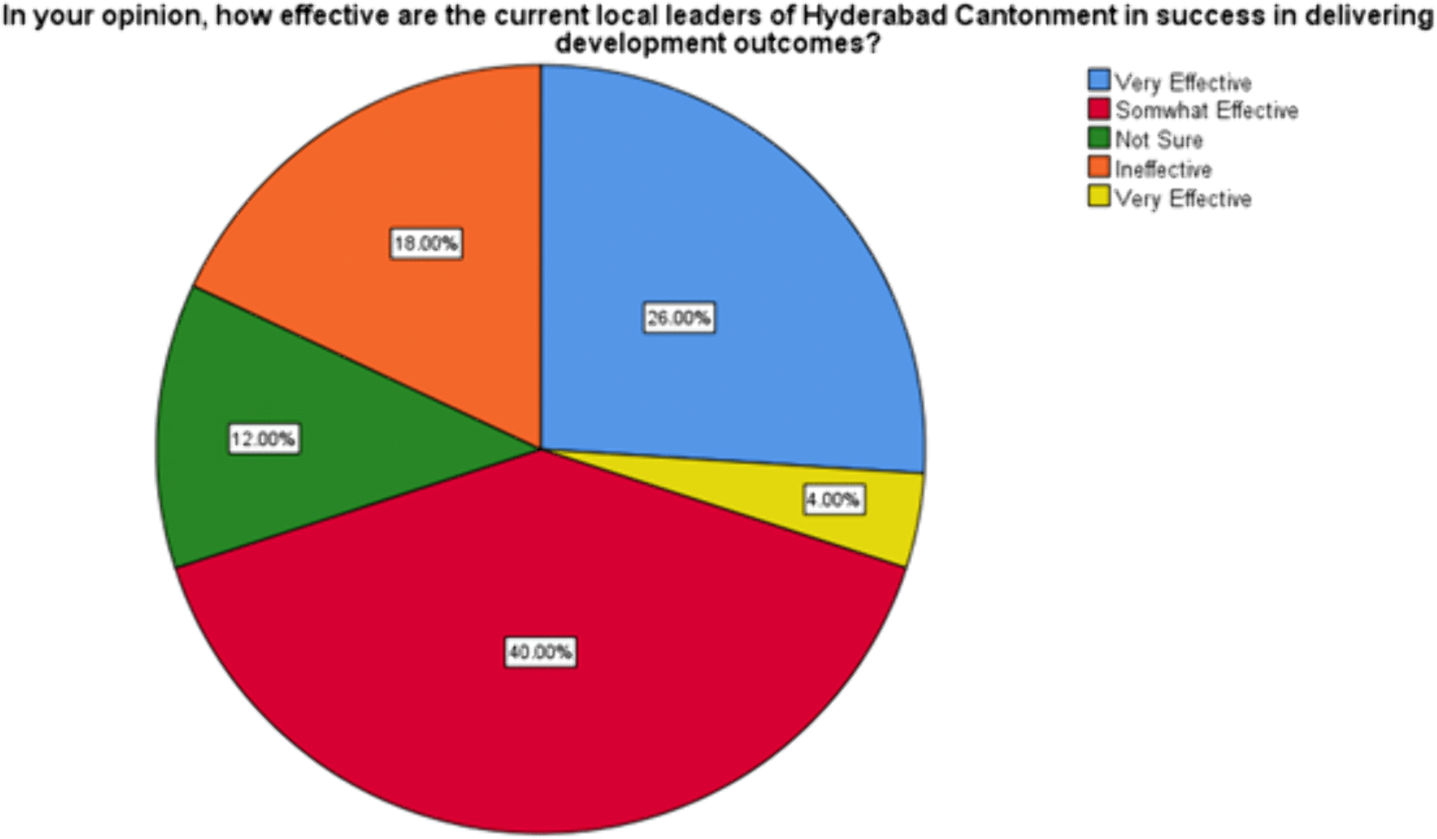
Perceived effectiveness of current local leadership in delivering development outcomes.

Fig 1 shows that different demographic groups have rather different opinions about how effective local leadership is. Younger respondents and those with moderate levels of education are comparatively more positive toward local administrative performance, whereas highly educated respondents demonstrate more critical attitudes, indicating higher expectations regarding governance quality and development outcomes. These findings suggest that demographic characteristics significantly influence public perceptions of leadership effectiveness.

#### Achievement of promised community development goals.

**Fig 2:**
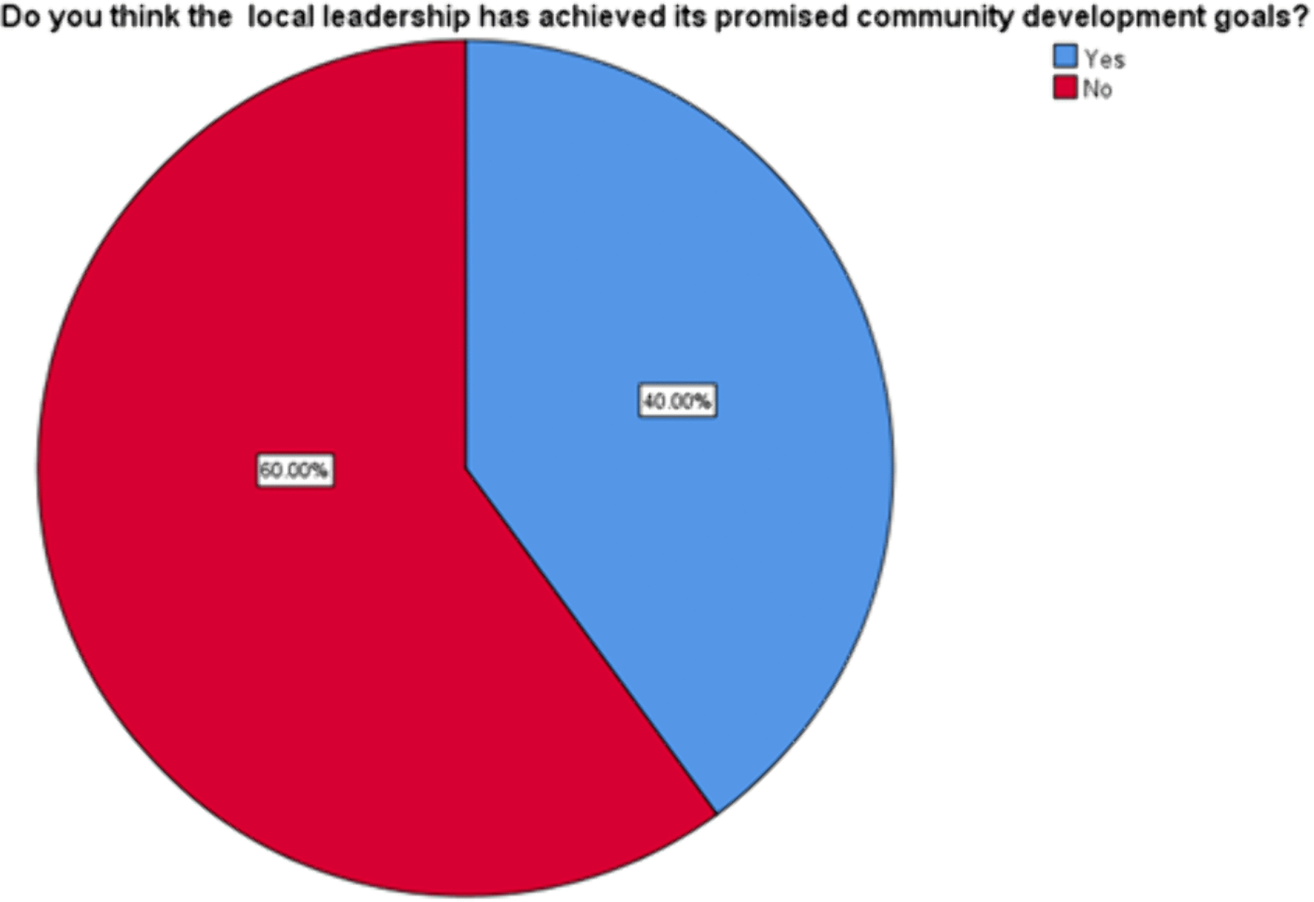
Perceptions regarding achievement of promised community development goals.

Fig 2 indicates that opinions vary considerably regarding whether local leaders fulfilled their development promises. Respondents with lower educational attainment tend to express relatively more satisfaction, whereas graduate-level respondents exhibit greater dissatisfaction with development outcomes. These trends imply that evaluative judgments become increasingly critical with higher educational exposure and political awareness.

#### Factors leading to poor performance of local leaders.

**Fig 3:**
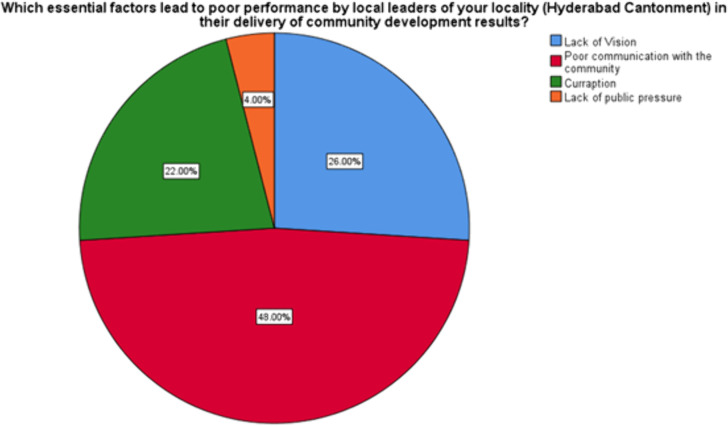
Factors contributing to poor leadership performance.

Fig 3 demonstrates that respondents attribute poor leadership performance mainly to lack of vision and weak communication with the community. Respondents with higher educational backgrounds are particularly critical regarding communication failures, whereas less educated groups emphasize inadequate strategic direction. Corruption was comparatively less emphasized, suggesting that institutional inefficiencies are viewed as more significant barriers than intentional malpractice.

#### Most important factor in poor local development.

**Fig 4:**
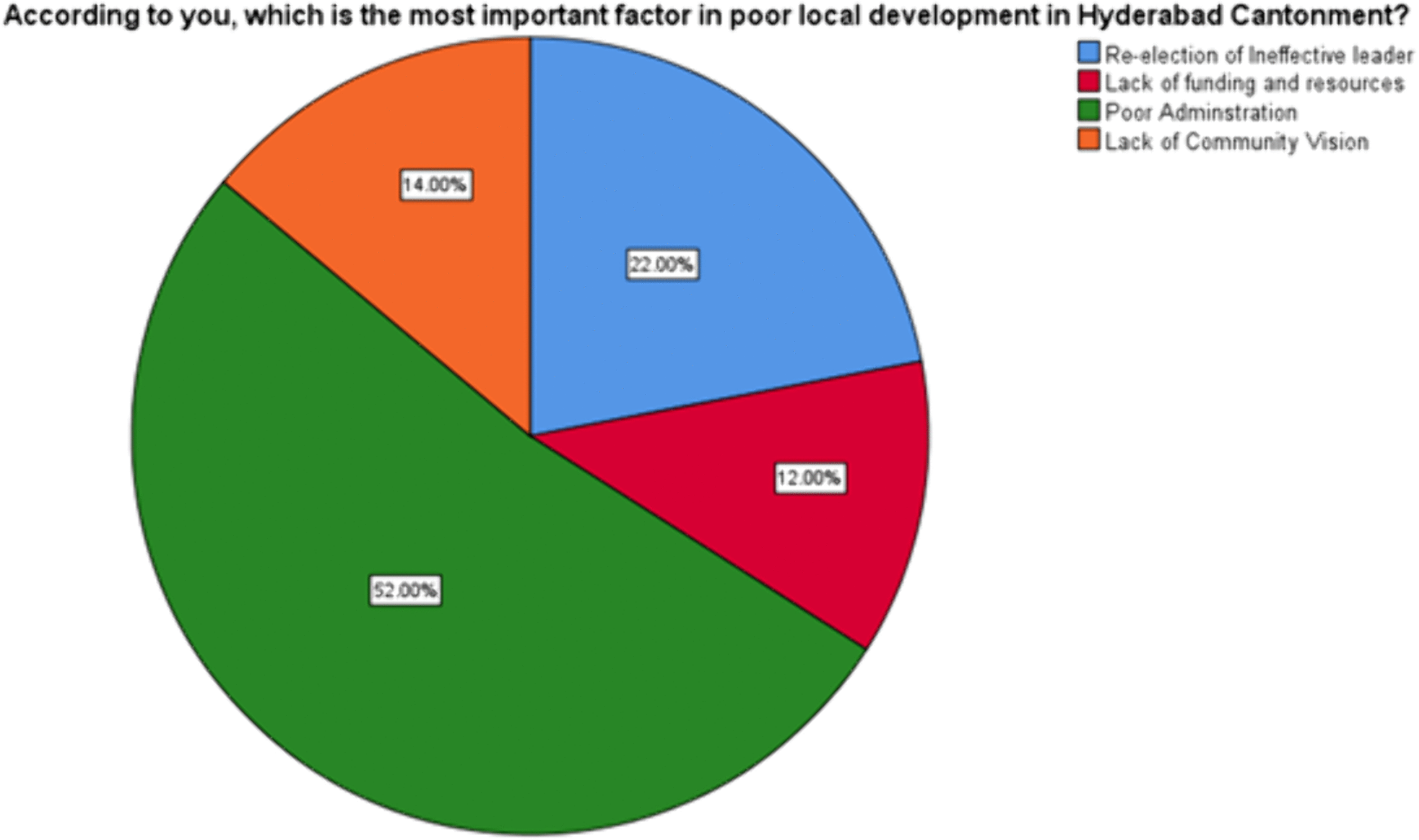
Most important factor contributing to poor local development.

Fig 4 reveals that respondents predominantly associate local underdevelopment with administrative weaknesses and governance-related inefficiencies. Middle-aged and educated participants especially identify poor administration as a major challenge, whereas younger respondents frequently emphasize weak leadership continuity. These findings indicate that governance-related shortcomings are considered more influential than financial limitations alone.

#### Impact of re-electing the same leaders.

**Fig 5:**
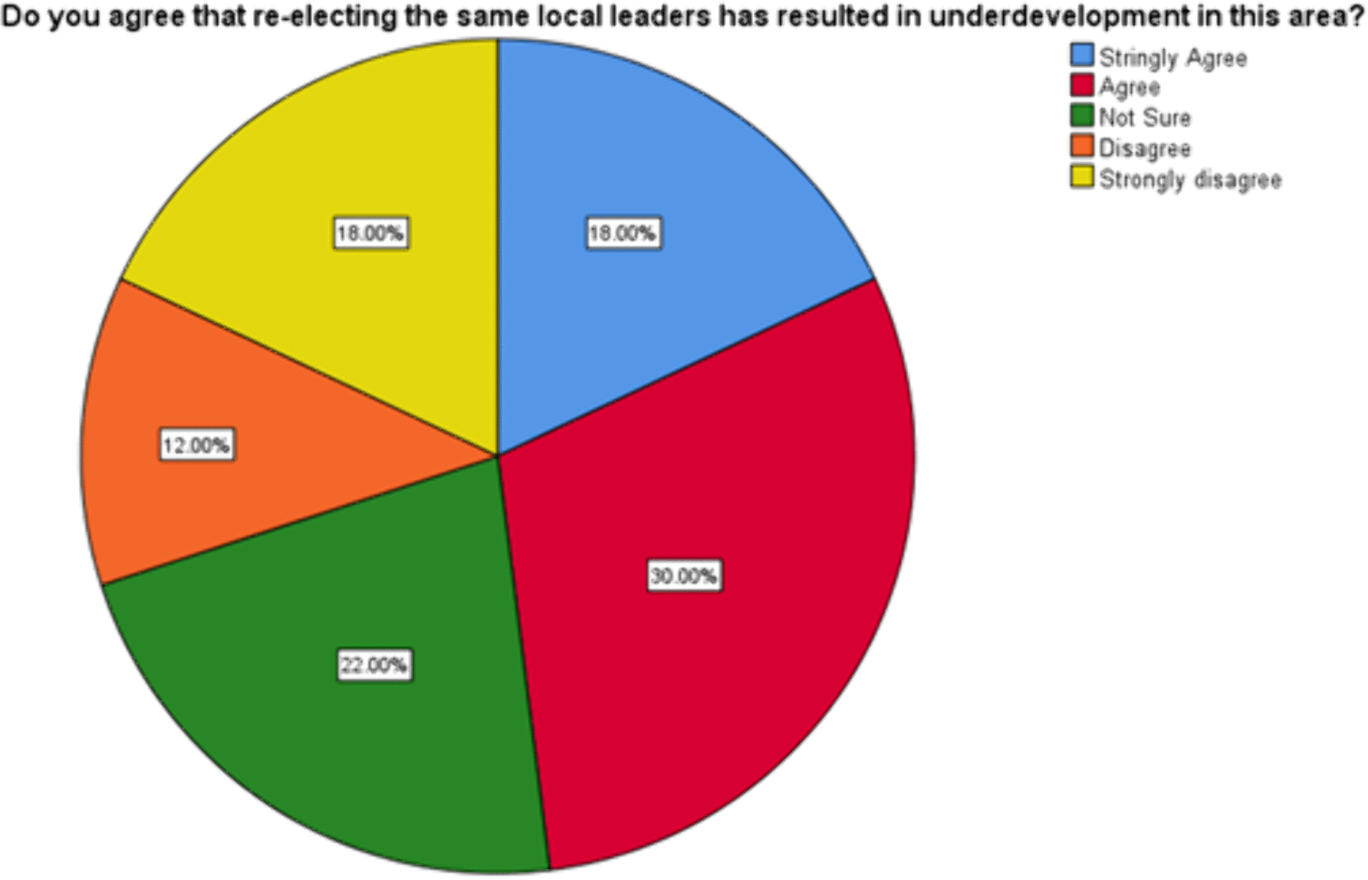
Perceived impact of re-electing the same local leaders.

Fig 5 demonstrates that many respondents believe repeated election of the same leadership contributes to continued underdevelopment. Agreement is particularly strong among younger and less educated groups, whereas more educated respondents present comparatively nuanced opinions. These patterns indicate that political continuity is frequently associated with dissatisfaction regarding development performance.

#### Importance of cooperation between leadership levels.

**Fig 6:**
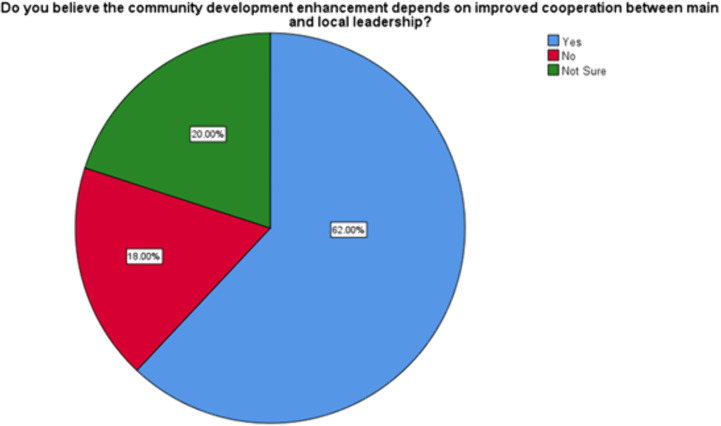
Importance of cooperation between main and local leadership.

Fig 6 indicates broad agreement that effective community development depends on cooperation between local and higher-level leadership structures. Educated and experienced respondents particularly recognize the importance of institutional coordination mechanisms in ensuring sustainable development outcomes.

#### Types of support lacking from higher leadership.

**Fig 7:**
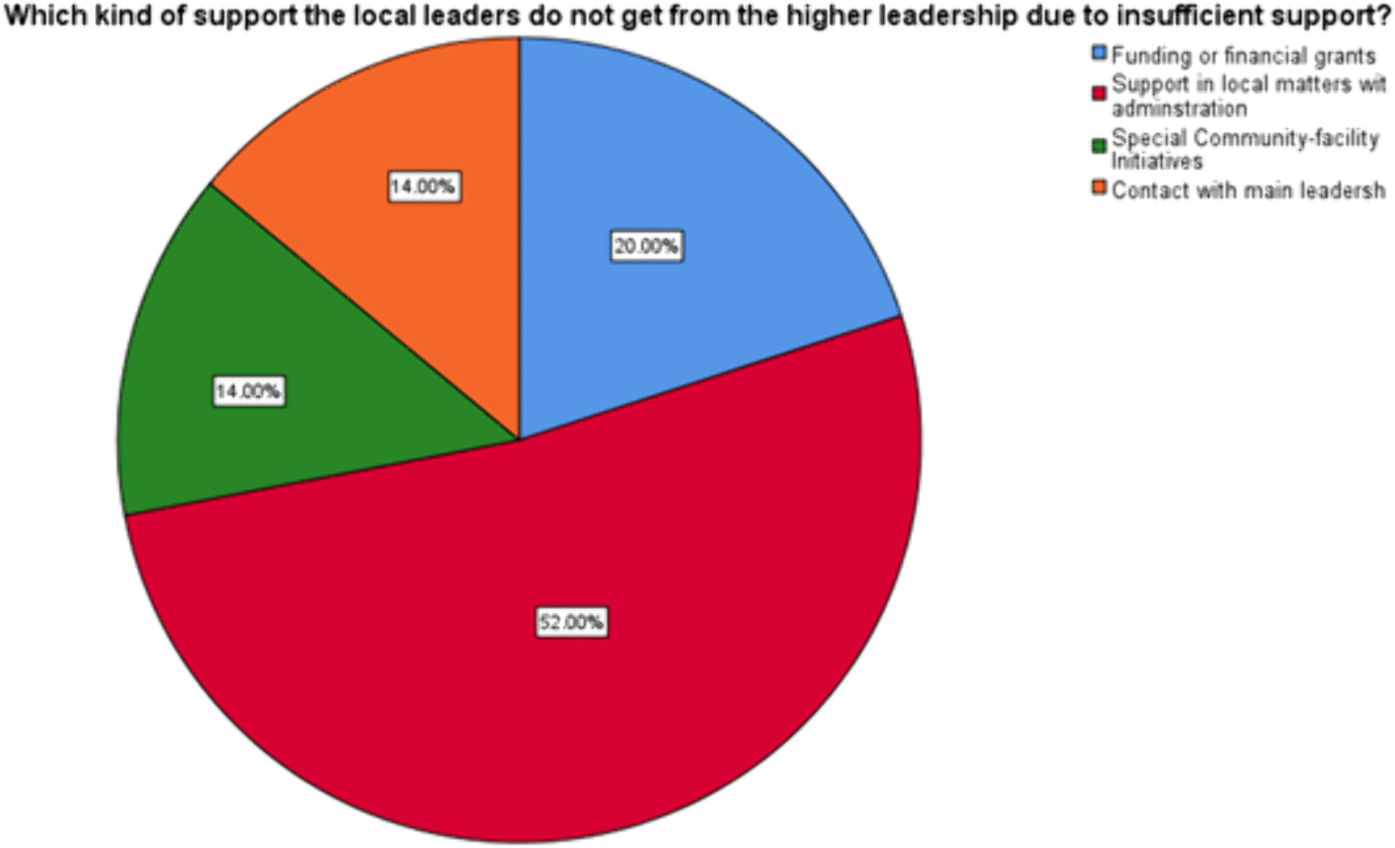
Support deficiencies from higher leadership authorities.

Fig 7 illustrates that respondents most frequently identify financial and administrative assistance as insufficiently provided by higher authorities. Younger respondents additionally emphasize weak coordination and communication between leadership levels. These perceptions reflect dissatisfaction with institutional support mechanisms affecting local development initiatives.

#### Failure to acquire funding for development.

**Fig 8:**
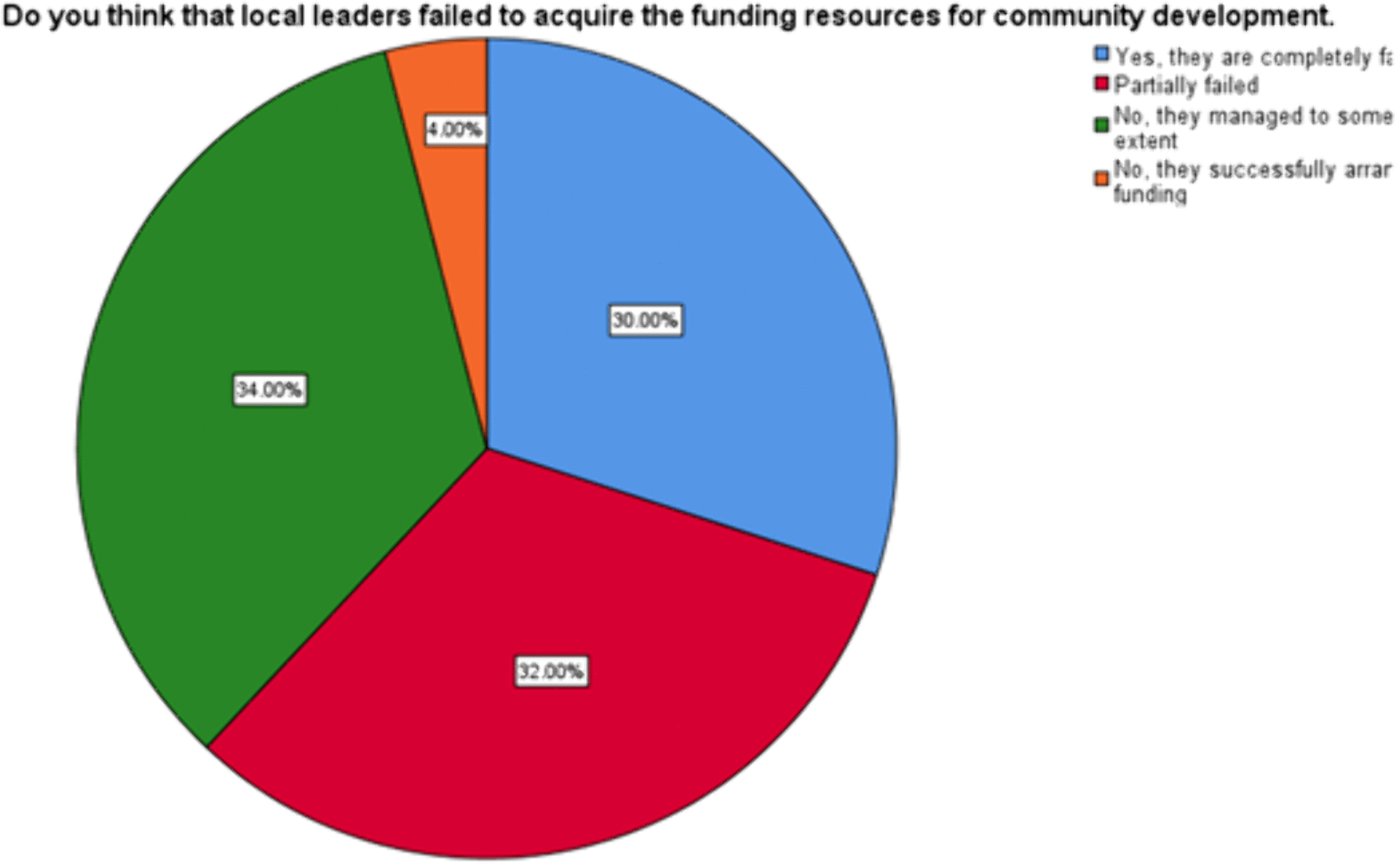
Perceived failure in acquiring development funding.

Fig 8 shows that respondents generally believe local leadership has struggled to secure adequate development funding. Younger respondents particularly perceive substantial shortcomings in resource mobilization, while highly educated respondents acknowledge only partial success. These results suggest that ineffective resource acquisition significantly influences public evaluations of leadership performance.

#### Perceived benefit of NGO involvement.

**Fig 9:**
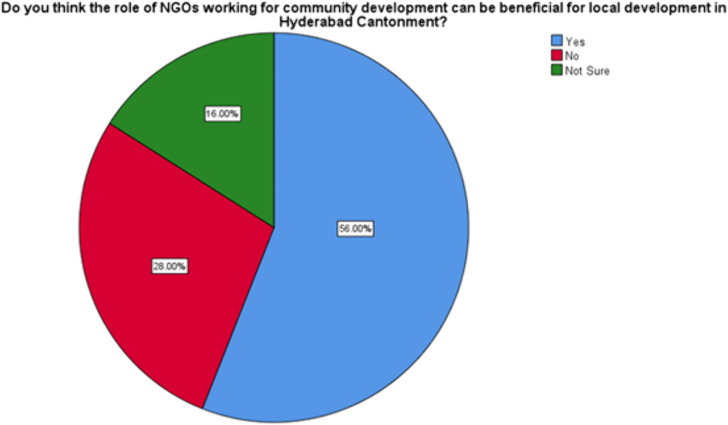
Perceived benefits of NGO participation in local development.

Fig 9 demonstrates that most respondents view NGOs as important contributors to community development. Younger and middle-aged participants particularly support NGO involvement, indicating acceptance of collaborative and participatory development approaches. Overall, NGOs are largely perceived as complementary actors capable of strengthening grassroots initiatives.

#### Perceptions of women’s leadership effectiveness.

**Fig 10:**
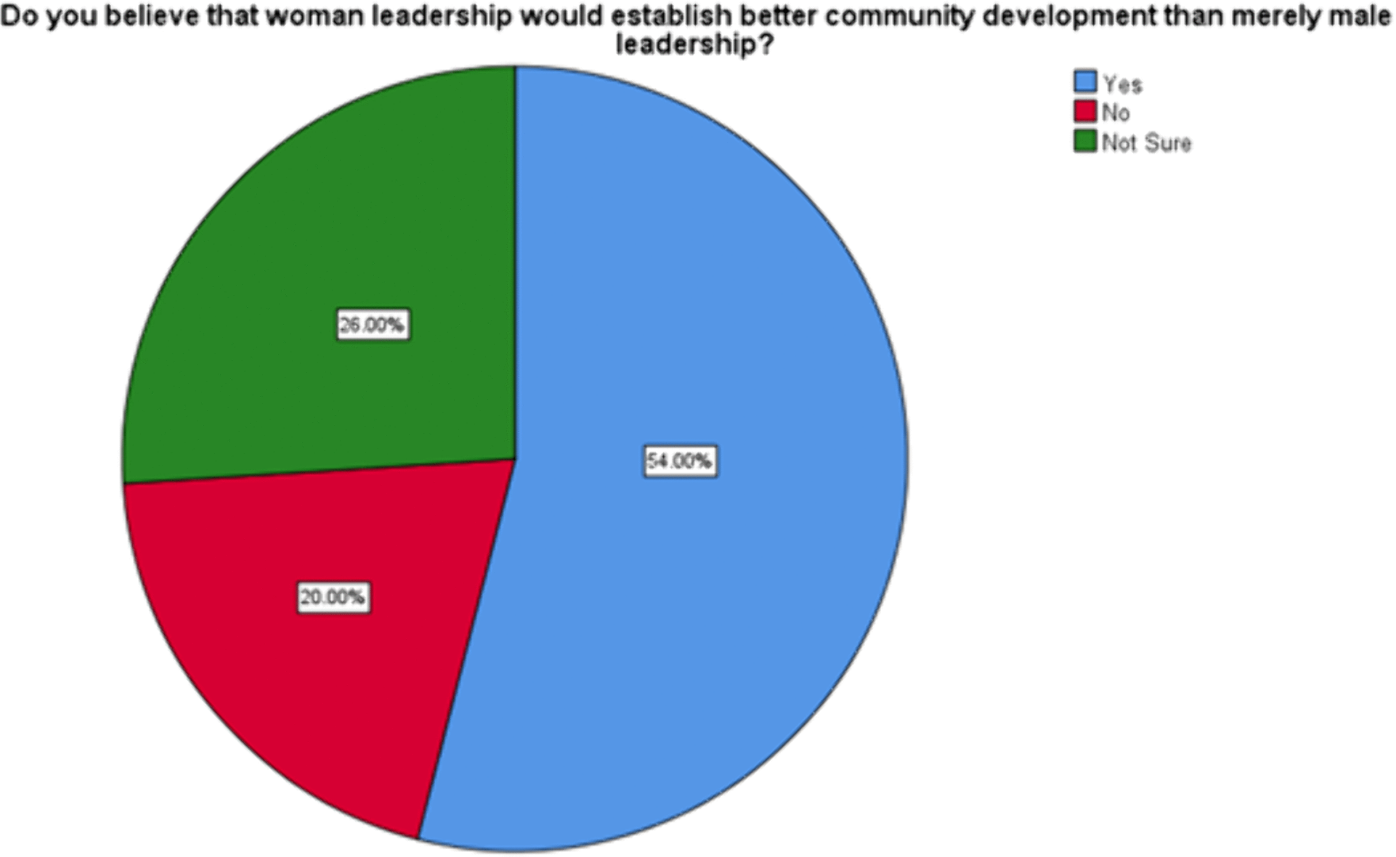
Perceptions regarding women’s leadership effectiveness.

Fig 10 indicates generally positive perceptions regarding the role of women in leadership. Many respondents believe women leaders can contribute effectively to inclusive governance and community development outcomes. Although skepticism remains among some older and conservative participants, the broader trend reflects increasing acceptance of gender-inclusive leadership structures.

Overall, the graphical evidence presented in Figs 1-10 consistently supports the broader analytical conclusions of the study. The figures collectively demonstrate strong relationships between perceptions of leadership effectiveness, institutional coordination, resource mobilization, and gender inclusion. These quantitative patterns complement the qualitative findings and reinforce the argument that sustainable local development in Hyderabad Cantonment requires inclusive, transparent, and community-oriented leadership reforms.

### Hypothesis testing

The following inferential analyses are presented as exploratory statistical assessments intended to complement the qualitative findings. Given the non-probabilistic sampling strategy and limited sample size, the results should be interpreted as indicative rather than confirmatory.

***RQ***_**1**_ **: How far leadership as a characteristic of Community Capacity Building (CCB) contribute to promoting local development in Hyderabad Cantonment?**

- Null Hypothesis (*H*0_1_): Local leadership in Hyderabad Cantonment is effective in delivering community development outcomes.
- Alternative Hypothesis (*H*1_1_): Local leadership in Hyderabad Cantonment is ineffective in delivering community development outcomes.

The findings in Table 2 provide strong evidence against the null hypothesis, with a t-value of 32.922, degrees of freedom (df) = 49, and a significance level (p-value) of.000. Because the p-value is less than 0.05, the alternative hypothesis is accepted and the null hypothesis is rejected. This implies that the local leadership of Hyderabad Cantonment is viewed as incapable of accomplishing the objectives of community development. The statistically significant result highlights a critical area for improvement by revealing a significant gap between leadership efforts and community expectations or needs within the broader framework of Community Capacity Building (CCB) to promote sustainable local development.

**Table 2:** Hypothesis testing result of *RQ*_1._

	t	df	Sig. (2-tailed)
*RQ*_1_	32.922	49	.000

***RQ***_**2**_ **: How does leadership influence participatory governance and community decision-making?**

- Null Hypothesis (*H*0_2_): Leadership does not have a significant influence on participatory governance and community decision-making in Hyderabad Cantonment.
- Alternative Hypothesis (*H*1_2_): Leadership has a significant influence on participatory governance and community decision-making in Hyderabad Cantonment.

The statistical findings in Table 3 reveal a t-value of 29.117 with 49 degrees of freedom and a significance level (p-value) of.000. Since the p-value is quite lower than the standard cutoff value of 0.05, the alternative hypothesis is accepted. This demonstrates that leadership statistically has a significant influence on community decision-making and participatory governance in Hyderabad Cantonment. The conclusion provides details on the importance of good leadership in promoting inclusive decision making, transparency, and community participation, which are all willing to enhance good governance and long term local development results.

**Table 3:** Hypothesis testing result of *RQ*_2._

	t	df	Sig. (2-tailed)
*RQ*_2_	29.117	49	.000

***RQ***_**3**_ **: To investigate the leadership contribution in the mobilization of resources and infrastructure development in Hyderabad Cantonment.**

- Null Hypothesis (*H*0_3_): Local leaders in Hyderabad Cantonment have been successful in acquiring sufficient funding and resources from the community and higher forums.
- Alternative Hypothesis (*H*1_3_): Local leaders in Hyderabad Cantonment have failed to acquire sufficient funding and resources from the community and higher forums.

The t-test result in Table 4 displays a t-value of 28.454 with 49 degrees of freedom and a significance level (p-value) of.000. Since the p-value is significantly less than 0.05, the null hypothesis is rejected, supporting the alternative hypothesis. This implies that local leaders in Hyderabad Cantonment have not been successful in obtaining sufficient funding and resources from the general public and higher authorities. Long-term community advancement and the implementation of sustainable development projects may be hampered by the findings, which indicate a leadership effectiveness gap in terms of infrastructure development and resource mobilization.

**Table 4:** Hypothesis testing result of *RQ*_3._

	t	df	Sig. (2-tailed)
*RQ*_3_	28.454	49	.000

***RQ***_**4**_ **: How do local community members perceive the effect of the absence of women leadership on community development?**

- Null Hypothesis (*H*0_4_): The absence of women in local leadership does not affect community development in Hyderabad Cantonment.
- Alternative Hypothesis (*H*1_4_): The absence of women in local leadership has a significant negative effect on community development in Hyderabad Cantonment.

The t-test result in Table 5 shows a t-value of 28.160, significantly below the 0.05 cutoff, with 49 degrees of freedom and a significance level of.000. As a result, the null hypothesis is rejected and the alternative hypothesis is accepted. The results indicate that the dearth of women in local leadership roles is believed to have a significant negative impact on the community development of Hyderabad Cantonment. It is known to the community that inclusive leadership, especially women participation, is the key to equal growth, social sensitivity, and more representative decision-making in the local system of governance.

**Table 5:** Hypothesis testing result of *RQ*_4._

	t	df	Sig. (2-tailed)
*RQ*_4_	28.160	49	.000

## Discussion

To illustrate how leadership functions within Community Capacity Building (CCB) in Hyderabad Cantonment, this section integrates qualitative narratives with quantitative evidence to discuss the findings in relation to the research questions.

### RQ_1_: Contribution of leadership to local development

Results indicate that there is a divided perception regarding leadership effectiveness. Several respondents criticized leaders for lacking inclusiveness and for failing to address broader community needs such as infrastructure and public services, although some acknowledged that leaders were approachable and service-oriented. These findings were supported by survey results, which demonstrated that perceptions varied significantly according to age and educational background. Younger and less educated respondents were comparatively more optimistic, whereas older and more educated respondents expressed stronger criticism regarding weak communication, ineffective planning, and lack of long-term vision. The statistical findings associated with these perceptions should be interpreted cautiously because the study relied on a relatively small and non-probability sample.

### *RQ*_2_: Influence on participatory governance and decision-making

Leadership was found to be inconsistent in encouraging participatory governance practices. Interview findings revealed that leaders often lose engagement with the community, particularly marginalized groups such as women, after elections. Survey responses further supported this dissatisfaction by linking weak governance outcomes with the repeated election of underperforming leaders and insufficient administrative support.

### *RQ*_3_: Leadership and resource mobilization

The qualitative findings indicated that leadership lacked innovative funding strategies and demonstrated limited engagement with external institutions, which contributed to poor resource utilization and slow infrastructure development. Survey data showed that educated respondents were comparatively more optimistic regarding the role of businesses and NGOs in supporting development, although concerns remained regarding poor planning, political favoritism, and insufficient political commitment.

### *RQ*_4_: Perceptions of women in leadership

Both qualitative and quantitative findings reflected cautious optimism regarding women’s leadership. Respondents recognized the potential of women leaders to improve inclusiveness and strengthen community development outcomes despite persistent cultural and institutional barriers. Survey findings further indicated that younger respondents showed greater acceptance of women in leadership roles, whereas older and more conservative participants remained comparatively skeptical.

### Synthesis

The findings across all research questions lead to a common conclusion that leadership in Hyderabad Cantonment remains constrained by structural weaknesses, limited accountability, and insufficient inclusiveness. Although respondents acknowledged the importance of leadership in promoting local development, the findings also exposed systemic inequalities through weak participatory governance, inefficient resource mobilization, and the under-representation of women in leadership roles.

### Methodological reflection and triangulation

The integration of qualitative and quantitative approaches strengthened the interpretive validity of the study through triangulation. Survey patterns were largely consistent with interview narratives regarding leadership accessibility, governance limitations, resource constraints, and lack of participation, thereby increasing confidence in the consistency of findings across both methods. However, this convergence should not be interpreted as evidence of causal relationships because both strands primarily relied on perception-based responses. In addition, the relatively small, non-random sample and gender imbalance limit the broader generalizability of the findings.

## Conclusion

This paper used a mixed-method approach to examine the role of leadership as a defining dimension of Community Capacity Building (CCB) in promoting community development in Hyderabad Cantonment. The integration of qualitative insights and quantitative survey findings enabled the study to capture both community perceptions and measurable patterns related to governance, inclusiveness, and resource mobilization.

The findings affirm that inclusive and effective leadership plays a critical role in achieving sustainable local development. However, the study also identified several institutional and administrative barriers, including weak coordination, limited strategic planning, and insufficient political support, which negatively affect leadership performance and community development outcomes. Although communities believe that leaders have the potential to mobilize resources, existing mechanisms are widely perceived as inadequate, while opportunities for collaboration with government institutions and external stakeholders remain underutilized.

The study further reveals that community participation in decision-making processes remains limited, particularly for women and marginalized groups, due to traditional power structures and restricted participatory mechanisms. The under-representation of women in leadership emerged as a recurring theme throughout the findings. Despite institutional and cultural challenges, respondents increasingly recognized the potential effectiveness of women leaders in promoting community development. Younger and better-educated respondents, in particular, expressed stronger support for inclusive and gender-balanced leadership models, indicating a gradual shift in public attitudes toward participatory governance.

At the same time, the findings of this study should be interpreted cautiously because the research was conducted using a relatively small sample size and a non-probability sampling approach within a specific local context. Therefore, the results primarily reflect the perceptions of respondents from Hyderabad Cantonment and may not be generalized to broader populations or other administrative settings. Nevertheless, the study contributes valuable contextual evidence to the growing literature on Community Capacity Building and local leadership in developing communities.

Future research should employ larger and probabilistic samples across multiple regions to improve the generalizability of findings and provide comparative insights into the relationship between leadership, inclusiveness, and sustainable community development.

## Limitations

This study was conducted specifically within Hyderabad Cantonment using a relatively small non-probability sample, therefore, the findings primarily reflect the perceptions of respondents within this localized administrative and socio-political context. The sampling strategy limits statistical representativeness and restricts the broader generalizability of the results to other urban communities, cantonment settings, or wider Pakistani populations. In addition, the study relied primarily on perception-based responses rather than objective governance performance indicators. Consequently, the findings should be interpreted as exploratory and context-specific analytical insights. Future studies using larger probabilistic samples, comparative regional designs, and objective development indicators are recommended to improve external validity and strengthen causal interpretation.

### Recommendations

- Build capacity in leadership at various levels of governance.
- Formalize gender inclusion mechanisms of representation.
- Empower collaboration with the civil society.
- Enhance horizontal coordination between the levels of administration.
- Introduce regular external auditing of the accounts
- Form participatory committees with the youth and women.
